# Spontaneous Spinal Subdural Hematoma Associated With Rivaroxaban and Aspirin Use: A Report of a Rare Case

**DOI:** 10.7759/cureus.70525

**Published:** 2024-09-30

**Authors:** Abner A Limardo, José J Berrios, Adrián Pagán, Reynaldo de Jesús, Rafael Espinet

**Affiliations:** 1 Graduate Medical Education, Centro Médico Episcopal San Lucas, Ponce, PRI; 2 Internal Medicine, Centro Médico Episcopal San Lucas, Ponce, PRI; 3 Neurosurgery, Centro Médico Episcopal San Lucas, Ponce, PRI

**Keywords:** bleeding risk, emergency neurosurgery, neurological deficit, risk factors, spontaneous spinal subdural hematoma

## Abstract

A spontaneous spinal subdural hematoma (SSSH) is a rare but potentially deadly condition characterized by the presence of blood in the subdural space, commonly causing compression of the spinal cord and acute neurological deficits. Urgent surgical intervention with a decompressive laminectomy is warranted to avoid lasting deficits. The literature on this pathology is scarce, and the etiology is still poorly understood, although associations have been established with arteriovenous malformations, rupture of epidural vessels, and anticoagulant use, among others.

This study presents the case of an 81-year-old Hispanic woman with a past medical history including an unspecified arrhythmia on treatment with oral anticoagulation who presented to the emergency room with sudden-onset, localized low back pain and an acute neurological deficit consisting of bilateral lower extremity paraplegia and areflexia. Magnetic resonance imaging (MRI) of the thoracic spine shows a 10-cm-long subdural hematoma causing compression from T5 to T10. At this time, the patient also developed an unstable atrial fibrillation with rapid ventricular response. After cardiac stabilization, a thoracic decompressive laminectomy with hematoma evacuation was performed.

The degree of preoperative neural deficit and time to surgical intervention are prognostic factors for clinical recovery in these patients. Anticoagulant medication use is a risk factor for SSSH, and a high index of suspicion is needed for patients presenting with acute-onset back pain and neurological deficits, especially in the setting of known risk factors. MRI is the diagnostic tool of choice, and urgent surgical decompression is warranted to prevent further neurologic deterioration.

## Introduction

A spontaneous spinal subdural hematoma (SSSH) is a rare but potentially deadly condition characterized by the presence of blood in the subdural space, commonly causing compression of the spinal cord and acute neurological deficits [1]. Its incidence is extremely rare, and therefore, the literature on the topic is still scarce [2-5]. Despite this, studies have proved associations with the presence of arteriovenous malformations, coagulopathies, and anticoagulant use [6-8]. The clinical picture usually consists of the acute onset of severe back pain that can rapidly progress to include neurological deficits [5,9]. Diagnosis is made with magnetic resonance imaging (MRI) of the affected area, and treatment involves urgent surgical decompression to avoid progressive and possibly permanent neurological decline [8].

## Case presentation

**Figure 1:**
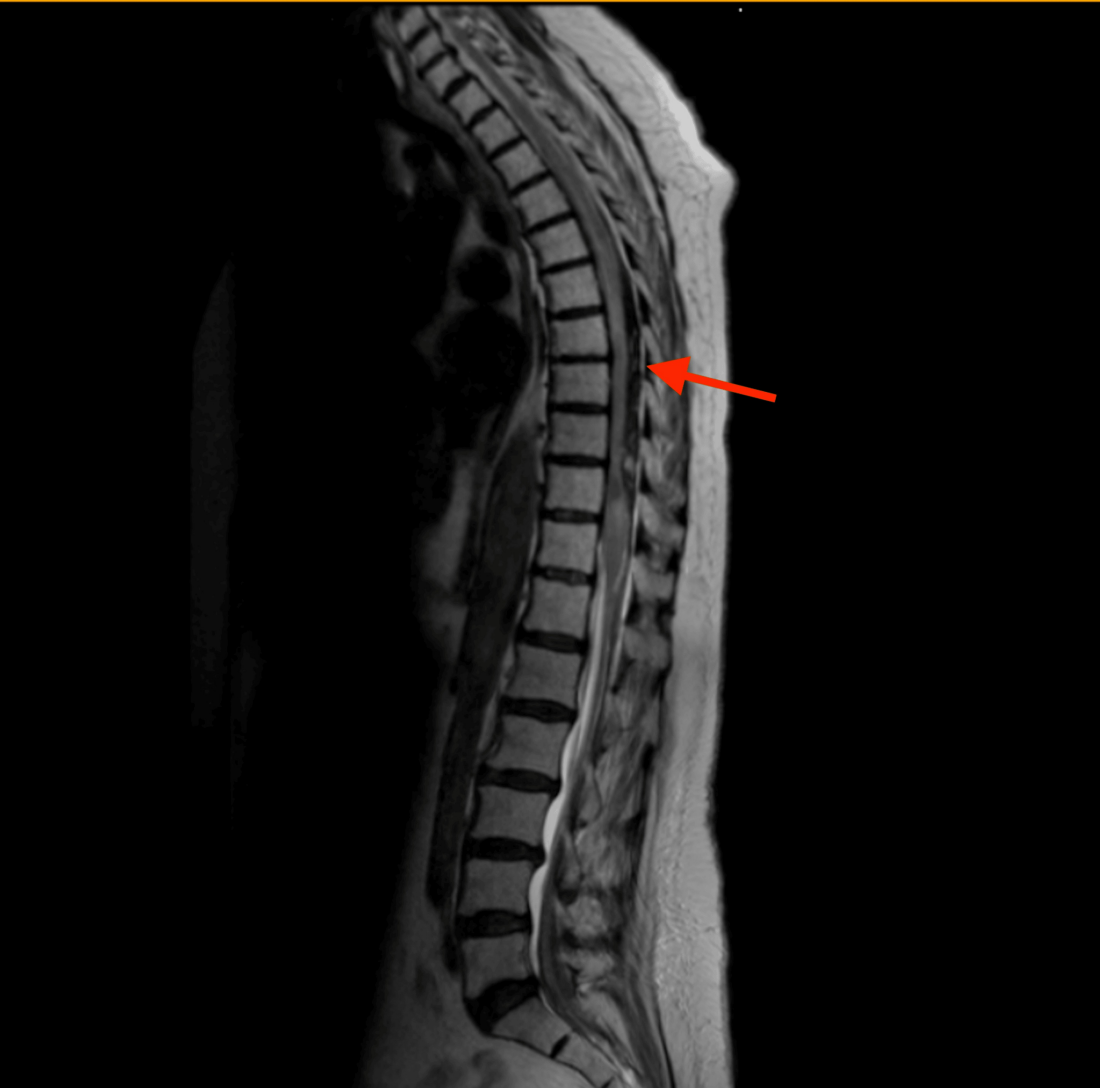
MRI of the thoracic spine showing 10 cm subdural hematoma MRI: magnetic resonance imaging

This study presents the case of an 81-year-old Hispanic woman with a past medical history including hypertension, coronary artery disease (CAD) with a previous myocardial infarction and two stent placements, and an unspecified arrhythmia for which a permanent pacemaker was implanted, among other comorbidities. The medication regimen included antiplatelet medication with aspirin and direct oral anticoagulant therapy with rivaroxaban. The patient presented to our emergency department with a chief complaint of intense, localized lower back pain that had started suddenly around 11 hours before. She denied any history of trauma and referred to bilateral lower extremity weakness with loss of sensation and an inability to ambulate. Vital signs were within normal limits. On physical examination, the patient was found with bilateral lower extremity flaccid paralysis, areflexia, absent anal tone, and anesthesia from the T8 dermatome downwards. Laboratory studies including complete blood count (CBC), basic metabolic panel (BMP), urinalysis, and coagulation profiles were within normal limits (Table 1). Initial imaging studies with computed tomography (CT) angiography showed no aneurysm, dissection, or any arteriovenous malformations, and thoracic and lumbar spine X-rays showed no identifiable fractures. Further workup with an MRI of the thoracic spine showed a 10-cm-long subdural hematoma causing compression from T5 to T10 (Figure 1). The patient was then scheduled with neurosurgery for urgent decompressive surgical intervention but went on to develop an unstable atrial fibrillation with rapid ventricular response. Due to the undeniable threat to the patient's life that this represented in the context of going under general anesthesia for surgical intervention, the surgery was postponed allowing for adequate cardiovascular optimization. Ultimately, after medical management and optimization, the patient's arrhythmia was stabilized, and a thoracic decompressive laminectomy with hematoma evacuation was successfully performed seven days after the patient's initial presentation to the emergency room. Additionally, the patient's subdural hematoma represented a contraindication to her long-term oral anticoagulant therapy being used for the control of atrial fibrillation. Therefore, the electrophysiology service was consulted, and the patient had a left atrial appendage closure device implanted for the prevention of future cardioembolic events. Currently, the patient is undergoing physical rehabilitation for paraplegia and coordination for home care services.

**Table 1:** Laboratory studies on admission

Laboratory study	Results	Normal range
Complete blood count		
White blood cells	10,510/µL	4,500-11,000/µL
Hemoglobin	14 g/dL	12.3-15.3 g/dL
Hematocrit	42%	35.9-44.6%
Platelet count	267,000/µL	150,000-450,000/µL
Basic metabolic panel		
Sodium	140 mEq/L	135-145 mEq/L
Potassium	4.9 mEq/L	3.5-5 mEq/L
Chloride	108 mEq/L	98-106 mEq/L
Carbon dioxide	24.2 mEq/L	23-30 mEq/L
Blood urea nitrogen	14 mg/dL	7-20 mg/dL
Creatinine	1.31 mg/dL	0.6-1.2 mg/dL
Glucose	152 mg/dL	70-140 mg/dL
Calcium	9.8 mg/dL	8.5-10.2 mg/dL
Coagulation studies		
Prothrombin time	13 seconds	11-13.5 seconds
Activated partial thromboplastin time	26 seconds	25-35 seconds
International normalized ratio	1.22	0.8-1.1
Urinalysis		
Urine color/appearance	Yellow	Light yellow to amber
Appearance	Clear	Clear
Urine pH	6.0	4.5-8.0
Urine-specific gravity	1.054	1.005-1.030
Urine protein, glucose, ketones, occult blood, nitrates, bilirubin, urobilinogen, leukocyte esterase, bacteria, ascorbic acid	Negative	Negative
Urine red blood cells	0-2/hpf	0-3/hpf
Urine white blood cells	0-2/hpf	0-5/hpf

## Discussion

A spinal subdural hematoma can result from trauma, occur as a rare complication of neuraxial anesthesia, or arise spontaneously (idiopathically) [4,10]. Patients often present in the fourth or fifth decade of life, and males are predominantly affected [4,9]. The etiology of this condition is still poorly understood, but multiple risk factors and associations have been made. These include arteriovenous malformations, anticoagulation and antiplatelet medication, and coagulopathies or blood dyscrasias. Although causality cannot be proved, our patient's aspirin and rivaroxaban use both represent a risk factor for SSSH due to impaired platelet and coagulation cascade function [6,11,12]. Due to the increase in the use of these medications for stroke prophylaxis and non-valvular atrial fibrillation, having a high index of suspicion for possible rare complications such as SSSH is of paramount importance for the adequate diagnosis and management of the condition [7].

Different mechanisms have been reported as the cause of bleeding in this condition such as the rupture of spinal veins or arteries or vascular malformations. An arterial source of bleeding has been proposed as a possible mechanism for patients with rapidly progressive neurological deficits, yet the most widely accepted theory outside of this scenario of rapid neurological symptom progression is a venous source of bleeding, and it is based on the anatomy in the region [1,2,6,13]. The internal epidural venous plexus drains the abdomen and thorax and is a low-pressure valveless venous system; thus, it is prone to rupture with changes in pressure. It is postulated that an increase in intra-abdominal pressure, such as with the Valsalva maneuver, transmits that increased pressure to the intraspinal segments of these vessels, which then rupture [2,6,9,12]. The existence of a "locus minoris resistentiae" within the epidural venous plexus is also proposed, consisting of a network of weakened epidural veins that are more prone to rupture with increases in pressure [3].

The classic clinical presentation associated with this condition is the acute onset of back or neck pain (depending on the level of the lesion) that can radiate to the extremities and is then followed by motor and sensory symptoms, usually flaccid paralysis with hyporeflexia [2,3,13]. A high variation in time has been reported between the onset of pain and the development of neurological deficits, ranging from hours to days or even months in some cases. Because of this, symptoms can mimic other pathologies such as fracture, lumbar herniation, and mass lesions, among others. A high index of suspicion is needed to adequately identify and manage this condition because failure to do so can have devastating consequences such as permanent neurological damage or even death. MRI of the affected area is the gold standard for the diagnosis of this condition and should be considered in patients with acute pain and neurological deficits [2,14].

The consensus for the adequate management of SSSH is urgent surgical decompression with hematoma evacuation [3,4]. For patients with tolerable pain and no neurological deficits, conservative management has been reported as adequate [5,15]. There are two main factors that have been found to affect the patient's neurologic recovery after management: time from onset of symptoms to surgical decompression and the severity of preoperative neurological deficit. Studies have shown that neurological improvement was greatest in patients who had surgical intervention performed within 12 hours of symptom onset [15,16]. Moreover, it has been found that patients with fewer preoperative neurological deficits had improved recovery of function after treatment [5,8,16]. Because of this, early recognition and diagnosis are crucial to halt the progression of neurological deterioration and give the patient the best chance at a full recovery after surgical management.

In the case of our patient, she presented to the emergency room with complete motor and sensory loss of bilateral lower extremities (Frankel grade A). Lawton et al., while proving the association between preoperative neurological deficit and post-surgical recovery of neurological function, found that out of the patients presenting preoperatively with complete motor and sensory loss (Frankel grade A), only 25% of them were able to regain normal neurological function, while the other 75% had improvement in function after surgical management but did not return to baseline. In contrast, out of their study patients who presented with incomplete motor loss and ability to ambulate (Frankel grade D), 83% of them were able to accomplish a complete recovery of neurologic function after the surgical evacuation of the hematoma [8]. Another important factor to consider is the level of the lesion. Larger hematomas in areas with less cord space, like the thoracic spine as in our patient, are associated with worse outcomes [1]. Even though the patient presented to the emergency room quickly after symptomatic onset and adequate diagnosis was made through MRI, the time to surgical intervention was delayed by her development of unstable atrial fibrillation. In this situation, the threat to life took precedence over the urgency of surgical intervention. Along with the severe neurological deficit upon presentation and the level of lesion, these factors make complete neurological recovery uncertain. Despite this uncertainty, reports indicate that delayed neurological recovery after surgery is possible and ongoing rehabilitation remains crucial for her potential recovery [8,14].

## Conclusions

An SSSH is a rare but potentially life-threatening neurosurgical emergency that requires prompt diagnosis and management to avoid rapidly progressive neurological deficits that can potentially be permanent and, in some cases, even cause death. A high index of suspicion is required for patients presenting with sudden-onset, intense low back pain with associated acute neurologic deficits, especially in the setting of risk factors such as anticoagulant or antiplatelet medication use. Urgent decompressive surgical intervention is needed to avoid further neurological deterioration, especially in patients presenting with severe deficits.
